# Psychiatric diseases and dementia and their association with open-angle glaucoma in the total population of Stockholm

**DOI:** 10.1080/07853890.2022.2148735

**Published:** 2022-11-21

**Authors:** Per E. Wändell, Gunnar Ljunggren, Lars Wahlström, Axel Carl Carlsson

**Affiliations:** a Division of Family Medicine and Primary Care, Department of Neurobiology, Care Sciences and Society (NVS), Karolinska Institutet, Huddinge, Sweden; b Academic Primary Health Care Centre, Region Stockholm Region, Stockholm, Sweden; c Centre for Psychiatry Research, Karolinska Institutet, Stockholm, Sweden

**Keywords:** Administrative databases, primary care, gender differences, age differences, epidemiology

## Abstract

**Objective:**

Association between some somatic diseases and primary open-angle glaucoma (POAG) are well-known. We aimed to study psychiatric diseases and dementia and their association with POAG in the total population of Region Stockholm.

**Methods:**

All living individuals above 18 years of age who resided in Stockholm County, Sweden, on 1 January 2017 (*N* = 1,703,675) were included. Data were obtained from administrative regional data. We identified individuals with specified psychiatric disorders in the years 2010–2019, and further identified those with an incident diagnosis of POAG during 2012–2018. Analyses were performed by age-group and sex. We calculated odds ratios (ORs) with 95% confidence intervals (95% CI), adjusted for age and neighborhood socio-economic status for individuals with POAG, and used individuals without POAG as referents.

**Results:**

A total of 16,299 cases of POAG were identified, of whom 9204 were women and 7095 men. Adjusted OR (95% CI) for the risk of POAG was 0.653 (0.610–0.698) for women and 0.714 (0.656–0.778) for men with dementia, respectively. The OR for POAG was 0.478 (0.355–0.643) for women with psychosis, and 1.164 (1.105–1.227) for women with depression. A high neighbourhood socio-economic status was associated with a higher risk of POAG. Other associations were non-significant.

**Conclusion:**

The prevalence of newly diagnosed POAG was decreased in men and women with dementia, and in women with psychosis, which could be an underestimation, owing to lack of investigation, which warrants attention. The risk of POAG was increased in women with depression, which could be secondary to the glaucoma diagnosis.

**KEY MESSAGES**
- The prevalence of newly diagnosed glaucoma was decreased in men and women with dementia, and in women with psychosis. A lower prevalence of newly diagnosed glaucoma may be due to an underestimation, owing to a lack of investigation.
- The risk of glaucoma was increased in women with depression, which could be secondary to the glaucoma diagnosis.

## Introduction

Glaucoma is an eye disease that is contributing to the burden of low vision and blindness in the world. It has been estimated that 1 out of 15 blind people is blind as a consequence of glaucoma, with 2.1 million people being blind and 4.2 million being visually impaired by glaucoma in the world [1]. As glaucoma is a treatable disease, it is important to identify individuals early to prevent future visual impairment. The possible effects of visual impairment include influence on social participation [2], and quality of life [3,4], but also an increased mortality [5]. Due to the aging of the population globally, glaucoma is associated with a high and increasing burden to health care and to the global health [6]. Besides, in general, lower socio-economic status is associated with a higher burden of glaucoma [6]. Regarding the relationship between socio-economic key factors and prevalence of glaucoma, one study showed a non-linear relationship with increasing risk glaucoma and the highest levels of income and education [7].

The most common type of glaucoma is primary open-angle glaucoma (POAG) and constitutes to up to three quarters of all registered glaucoma cases [8]. The POAG prevalence differs with estimated prevalence rates among individuals above 40 years of age between 1.4% in Western Asia and up to 5.2% in the Caribbean. The prevalence in Europe lies in between these figures, i.e. at around 2.0% [9]. Estimates of POAG have been published in the Nordic countries: a study from Finland reported a prevalence of 4.5% of the population aged 30 years and above [10].

As glaucoma is one out of several diseases affecting elderly, the comorbidity pattern of patients with glaucoma is important, not only for ophthalmologists but also for physicians in other medical specialties [11]. POAG is associated with several somatic diseases, especially diabetes, hypertension, and cancer [12].

Regarding comorbid psychiatric diseases, glaucoma has been shown to be associated with a higher risk of bipolar disease, major depressive disorders, and psychosis [13]. A review confirmed that glaucoma is more common in individuals with depression [14], even if a study published later found no increased risk of depression and anxiety in patients with glaucoma [15]. Glaucoma has also been found to be associated with Alzheimer’s disease [16], however, the reversed association, i.e. if psychiatric diseases and dementia are associated with an increased risk of incident glaucoma is less often studied.

Women have been shown to be more affected by visual impairment in general [17,18]. Besides, the presence of psychiatric comorbidities differs between men and women. Thus, analyses categorized by sex are desirable.

Accordingly, the aim of the present study was to investigate the association between psychiatric disorders and dementia, and the risk of newly diagnosed POAG in the total population of Region Stockholm, stratified by sex. We also aimed to study the role of neighbourhood socio-economic status on the risk of POAG in individuals with dementia and psychiatric disorders, to identify potential groups that may need attention.

## Methods and study population

The present study was based on the total adult population in Region Stockholm, with over 2.2 million inhabitants, which is more than one-fifth of the entire population of Sweden. Region Stockholm includes densely populated urban areas, the capital city of Stockholm with many suburbs and smaller cities and towns. However, Region Stockholm also includes a large archipelago and some sparsely populated rural areas. In Sweden, most of the health care is tax-financed, and with different taxation in the separate regions of Sweden. In Region Stockholm nearly all consultations and diagnoses from the health-care system are registered and stored in a central regional database, the VAL database, except for a few private clinics [19]. Thus, diagnoses from primary care, specialist open care, as well as in-hospital care are included. Data from the VAL database are registered in the Swedish National Patient Register (NPR) at the Swedish National Board of Health and Welfare (NBHW), however not data from primary care. The validity and accuracy of the NPR has been reported to be high [20]. Since 1997, diagnoses are coded according to the 10th edition of WHO’s International Classification of Diseases (ICD-10).

### Design

We conducted a study with cross-sectional design, comparing the presence of POAG in individuals with and without psychiatric diagnoses and dementia. The diagnoses were clinically based and were obtained from the VAL databases. We had no possibility to check for diagnostic criteria in specific cases.

### Study population

We included all adults (individuals above 18 years of age) residing in Region Stockholm on 1 January 2017 (*N* = 1,703,675). From VAL, we extracted data on all healthcare consultations in primary care, specialized open care, and in-hospital care between 2010 and 2019. We then identified all individuals who had at least one registration of out-patient care or hospital stay, and a new diagnosis of POAG during 2012–2018. In parallel, we identified specified psychiatric diagnoses registered in the VAL database in Region Stockholm 2010–2019.

### Outcome

The ICD-code H40.1 (primary open-angle glaucoma) was used to identify all incident diagnoses of POAG.

### Exposures

As background, the following diagnoses (with ICD10-codes) were chosen: dementia (F00, F01, F03, G30, and G20), schizophrenia and other psychotic conditions (F20, F23, F25, F28, and F29), mania and bipolar disease (F30 and F31), depression (F32, F33, F34, F38, and F39), anxiety disorders (F40 and F41), and neurotic and stress-related disorders (F42 and F43).

### Socio-economic status

We used a software tool named Mosaic to categorize neighbourhood socio-economic status (NSES) into three levels, i.e. high, middle, or low, by postal codes. The Mosaic tool was originally developed to categorize consumers by a marketing company (Experian) to streamline sale activities. It uses a multivariate modelling including more than 400 variables and is suitable for epidemiologic research and includes data from 23 countries [21,22].

### Ethics

Management and analyses of data from the VAL database in Region Stockholm are continuously assessed in the quality control of the healthcare utilization. Ethical approval has been obtained from the Regional Ethical Review Board in Stockholm to study comorbidities with these data. All data are pseudonymized, and waiver for informed consent is approved by the Regional Ethical Review board in Stockholm.

### Statistical methods

We used standard descriptive statistics for describing the studied sample and population, stratified by sex. Logistic regression with odds ratios (OR) and 95% confidence intervals was calculated for the odds of being diagnosed with POAG in individuals with the presence of psychiatric disorders and dementia verses those without these specific diagnoses, with adjustment for age and neighbourhood socio-economic status. Statistical analysis and data management were performed using SAS software, version 9.3 (SAS Institute Inc., Cary, NC).

## Results

**Figure 1.**
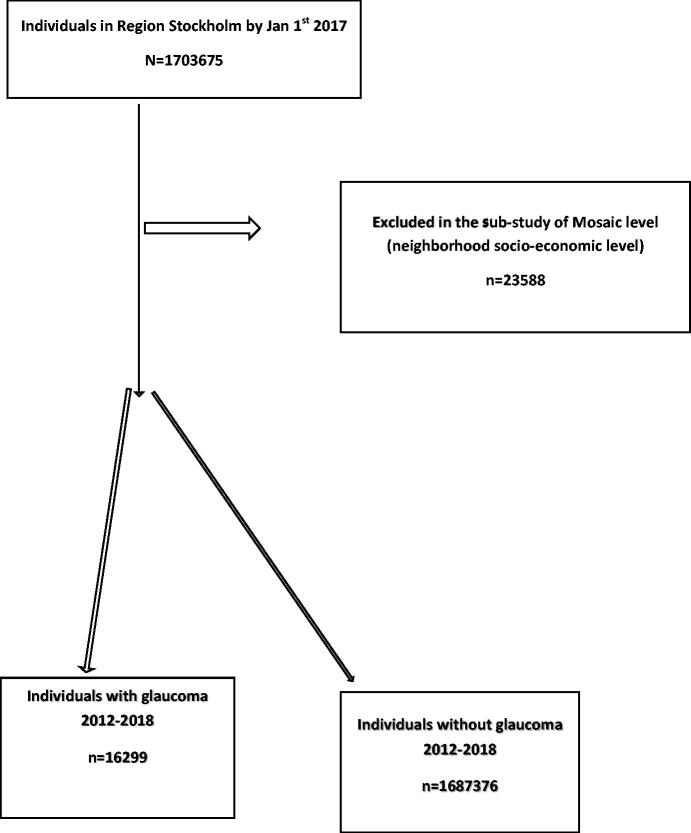
Flow chart of exclusion and inclusion of individuals, and categorization presence of primary-angle glaucoma or not.

In total 1,703,675 individuals (863,318 women and 840,357 men) living in Region Stockholm on 1 January 2017 were included in the study (Figure 1 and Table 1). Totally 16,299 individuals (9204 women and 7095 men) with newly diagnosed POAG during the years 2012–2018.

**Table 1.** Population (age 19 years of age and older) in Region Stockholm 1 January 2017, by age-groups and gender, with number of incident glaucoma, and by age-groups and socio-economic level by Mosaic.

Age group	Women		Men		All	Mosaic level			
	All	Glaucoma	All	Glaucoma		High level	Middle level	Low level	Missing data
	*n* (%)	*n* (%)	*n* (%)	*n* (%)	*n* (%)	*n* (%)	*n* (%)	*n* (%)	*n* (%)
									
19–49 years	502,683	198	514,898	312	1,017,581	454,807	195,098	350,151	17,525
	(58.23)	(2.15)	(61.27)	(4.40)	(59.73)	(44.69)	(19.17)	(34.41)	(1.72)
50–64 years	184,395	1114	184,922	1219	369,317	169,004	63,810	132,169	4334
	(21.36)	(12.10)	(22.01)	(17.18)	(21.68)	(45.76)	(17.28)	(35.79)	(1.17)
65–74 years	102,885	2835	92,505	2430	195,390	88,464	33,934	71,712	1280
	(11.92)	(30.80)	(11.01)	(34.25)	(11.47)	(45.28)	(17.37)	(36.70)	(0.66)
75–84 years	51,049	3029	37,961	2115	89,010	36,673	15,956	36,041	340
	(5.91)	(32.91)	(4.52)	(29.81)	(5.22)	(41.20)	(17.93)	(40.49)	(0.38)
85-w years	22,306	2028	10,071	1019	32,377	13,436	6760	12,072	109
	(2.58)	(22.03)	(1.20)	(14.36)	(1.90)	(41.50)	(20.88)	(37.29)	(0.34)
Total	863,318	9204	840,357	7095	1,703,675	762,384	315,558	602,145	23,588

Percentages by gender are shown vertically, and by Mosaic level shown horizontally (with missing data for Mosaic level).

The number of women and men with the studied psychiatric disorders and dementia by the presence of incident POAG or not is shown in Table 2. The ORs for POAG, in individuals with psychiatric diagnoses and dementia are shown in Table 3. A lower risk of POAG was found in men and women with a registered diagnosis of dementia, and in women with a diagnosis of psychosis. A higher risk of POAG was found in women with depression. The risk of POAG was higher in neighbourhoods with high socio-economic status compared to neighbourhoods with low socio-economic status (Table 3).

**Table 2.** Psychiatric comorbidity groups in the whole Stockholm population over the age of 18 years, in women and men separately, without and with incident glaucoma in 2012–2018.

Psychiatric disorders	Without glaucoma	With glaucoma	Population
	*n* (%)	*n* (%)	*n*
Women:			
Dementia	26,724 (2.71)	1,105 (12.01)	27,829
Psychosis	8,531 (0.87)	48 (0.52)	8579
Bipolar disorders	12,495 (1.27)	71 (0.77)	12,566
Depression	178,670 (18.12)	1,850 (20.10)	180,520
Anxiety	191,036 (19.37)	1,453 (15.79)	192,489
Neurotic and stress-related disorders	188,336 (19.10)	816 (8.87)	189,152
Total	605,792	5343	611,135
			
Men:			
Dementia	18,027 (1.85)	667 (9.40)	18,694
Psychosis	9585 (0.98)	43 (0.61)	9628
Bipolar disorders	7519 (0.77)	44 (0.62)	7563
Depression	98,142 (10.08)	786 (11.08)	98,928
Anxiety	101,367 (10.42)	568 (8.01)	101,935
Neurotic and stress-related disorders	79,644 (8.18)	340 (4.79)	79,984
Total	314,304	2448	316,752

Percentages denote out of the total numbers of women and men, respectively.

**Table 3.** Odds ratios (ORs), first for the association between psychiatric diseases and incident glaucoma during 2012–2018, with adjustment for age and neighborhood socio-economic status in men and women, separately; and second, the risk in three levels of neighborhood socio-economic status (NSES) of open-angle glaucoma in individuals with different for different psychiatric diseases and dementia during 2012–2018.

Psychiatric diseases	Women	Men	Women, NSES		Men, NSES	
	2012–2018	2012–2018				
			Highest versus lowest	Middle versus lowest	Highest versus lowest	Middle versus lowest
	OR (95% CI)	OR (95% CI)	OR (95% CI)	OR (95% CI)	OR (95% CI)	OR (95% CI)
Dementia	**0.65 (0.60–0.70)**	**0.71 (0.66–0.78)**	**1.080 (1.031–1.132)**	0.994 (0.937–1.055)	**1.082 (1.026–1.141)**	1.055 (0.985–1.130)
Psychosis	**0.48 (0.36–0.64)**	0.81 (0.60–1.10)	**1.075 (1.026–1.127)**	0.988 (0.931–1.049)	**1.082 (1.026–1.141)**	1.053 (0.983–1.127)
Bipolar disorders	1.04 (0.82–1.31)	1.03 (0.76–1.40)	**1.077 (1.028–1.128)**	0.989 (0.932–1.050)	**1.083 (1.027–1.142)**	1.053 (0.983–1.127)
Depression	**1.16 (1.11–1.23)**	1.03 (0.96–1.12)	**1.078 (1.029–1.130)**	0.989 (0.932–1.050)	**1.083 (1.027–1.142)**	1.053 (0.983–1.127)
Anxiety	1.05 (0.99–1.11)	1.03 (0.94–1.12)	**1.078 (1.028–1.129)**	0.989 (0.932–1.050)	**1.083 (1.027–1.142)**	1.053 (0.983–1.127)
Neurotic and stress-related disorders	0.97 (0.90–1.04)	1.09 (0.97–1.21)	**1.077 (1.028–1.128)**	0.989 (0.932–1.050)	**1.083 (1.027–1.142)**	1.053 (0.983–1.127)

Significant findings are shown in bold.

## Discussion

The most notable finding of this study was that we found a lower risk of incident POAG in individuals with dementia, and in women with psychosis.

Earlier studies have found an association between glaucoma and an increased risk of Alzheimer’s disease [16], and psychiatric disorders [13], even if one study could not confirm the presence of the increased risk [15]. In a recent Swedish study, no association between POAG and Alzheimer’s disease could be detected [23]. Glaucoma has also been described as a widespread neurodegenerative condition with similar pathogenetic mechanisms as for example Alzheimer’s disease [24,25]. In fact, the cholinergic system is important in the visual system, and anticholinergic medications have earlier been used to lower eye pressure in glaucoma, and systemic anticholinergic medications are indicated in Alzheimer’s disease [26]. A possible explanation for our findings is therefore that glaucoma is underdiagnosed in patients with dementia and psychosis [27,28]. A Finnish study showed that although visual impairment due to glaucoma had increased, the risk of visual impairment in patients treated for glaucoma decreases [29]. An alternative explanation is that medication in psychiatric disorders and Alzheimer’s disease could affect the risk of incident POAG. The side-effects of some psychotropic drugs for patients with angle-closure glaucoma is well known, with risk of achieving mydriasis and increased intra-ocular pressure [30].

The increased risk of depression in women with glaucoma could thus also possibly be associated to use of antidepressant drugs [30]. Glaucoma is associated with depression [14], although the health-related quality of life in glaucoma patients has found to be good in the absence of visual impairment [31], why the reason for a depression diagnosis in patients with glaucoma is not easily explained.

The possible explanation to our findings with glaucoma probably being underdiagnosed in patients with dementia and psychosis is of interest to be discussed further [27,28]. As almost all consultations in Region Stockholm are reported in the VAL-database, the low numbers of POAG diagnoses for individuals with dementia and psychosis do indicate that these individuals have not been examined for possible glaucoma. These individuals are vulnerable, and for example the risk of cardiovascular death in individuals with severe mental illness have doubled since the 1990s [32]. Screening for CVD has been shown to be low in such patients [32,33]. As glaucoma with possible visual impairment as a consequence can affect the health an easy examination such as an eye pressure measurement may certainly do a difference. We have earlier found prevalence of hypertension to be high in men with psychosis [12], in line with results from a review [34].

We found an increased risk of POAG in individuals living in high socio-economic status areas, in comparison to individuals living in low socio-economic status areas. In general, lower socio-economic status is associated with a higher burden of glaucoma [6]. Regarding the relationship between socio-economic key factors and prevalence of glaucoma, one study showed a non-linear relationship with increasing risk glaucoma and the highest levels of income and education [7]. The higher risk may be explained by a higher awareness of glaucoma in individuals with higher education and income. Thus, there seems to be a paradox with a higher risk of glaucoma in the highest socio-economic level, as lower socio-economic status in general is associated with higher morbidity and mortality [35,36]. Such paradoxes have also been described earlier for other diseases, e.g. a higher dementia risk in individuals with atrial fibrillation living in high socio-economic status areas [37].

This study has limitations. Individuals with POAG might have been missed. The possibility to identify patients with glaucoma has been estimated in various studies. The sensitivity of a glaucoma diagnosis in a Canadian study was estimated to be 76% [38], and similarly in the UK, it was estimated that up to as much as two-thirds of all POAG patients were undetected [39]. There might also be problems with overdiagnosis of glaucoma [40]. Earlier similar studies as the present have mostly restricted the diagnostic window to include only POAG H40.1 [41], while others also have included ocular hypertension (OHT), i.e. H40.0A [42]. We were recommended to include only a diagnosis of POAG, as the OHT group actually has no definite glaucoma diagnosis. Furthermore, we used logistic regression for the separate years and merged the results. We could not identify the first occasion of a diagnosis of the included psychiatric diseases, which was the reason not to use Cox regression analysis. Strengths of the present study include the high quality of Swedish registers [20,43]. Besides, data from the VAL-register in Region Stockholm have earlier been used in several studies, even if no formal validation study has been performed [19,44].

In conclusion, we found depression to be associated with a higher risk of POAG, which may be secondary to a glaucoma diagnosis. We found dementia in both men and women, and psychosis in women, to be associated with a lower risk of incident POAG. It is of importance to be aware of the increased risk of glaucoma going undetected in the care of these patients, as well as in individuals residing in areas with low socio-economic status.

## Data Availability

All data upon which the present manuscript is based, can be obtained from halsodata.rst@regionstockholm.se. Ethical permits are required. The authors of the manuscript are not allowed to share their data due to GDPR.
